# TaARPC3, Contributes to Wheat Resistance against the Stripe Rust Fungus

**DOI:** 10.3389/fpls.2017.01245

**Published:** 2017-07-18

**Authors:** Tuo Qi, Juan Wang, Qixiong Sun, Brad Day, Jun Guo, Qing Ma

**Affiliations:** ^1^State Key Laboratory of Crop Stress Biology for Arid Areas, College of Plant Protection, Northwest A&F University Yangling, China; ^2^Department of Plant, Soil and Microbial Sciences, Michigan State University, East Lansing MI, United States; ^3^Plant Resilience Institute, Michigan State University, East Lansing MI, United States

**Keywords:** ARPC3, cytoskeleton, wheat, stripe rust, yeast mutant, virus-induced gene silencing (VIGS)

## Abstract

The actin cytoskeleton participates in numerous cellular processes, including less-characterized processes, such as nuclear organization, chromatin remodeling, transcription, and signal transduction. As a key regulator of actin cytoskeletal dynamics, the actin related protein 2/3 complex (Arp2/3 complex) controls multiple developmental processes in a variety of tissues and cell types. To date, the role of the Arp2/3 complex in plant disease resistance signaling is largely unknown. Herein, we identified and characterized wheat ARPC3, *TaARPC3*, which encodes the C3 subunit of the Arp2/3 complex. Expression of *TaARPC3* in the *arc18* mutant of *Saccharomyces cerevisiae* Δ*arc18* resulted in complementation of stress-induced phenotypes in *S. cerevisiae*, as well as restore wild-type cell shape malformations. TaARPC3 was found predominantly to be localized in the nucleus and cytoplasm when expressed transiently in wheat protoplast. *TaARPC3* was significantly induced in response to avirulent race of *Puccinia striiformis* f. sp. *tritici* (*Pst*). Knock-down of *TaARPC3* by virus-induced gene silencing resulted in a reduction of resistance against *Pst* through a specific reduction in actin cytoskeletal organization. Interestingly, this reduction was found to coincide with a block in reactive oxygen species (ROS) accumulation, the hypersensitive response (HR), an increase in *TaCAT1* mRNA accumulation, and the growth of *Pst*. Taken together, these findings suggest that *TaARPC3* is a key subunit of the Arp2/3 complex which is required for wheat resistance against *Pst*, a process that is associated with the regulation of the actin cytoskeleton.

## Introduction

The plant actin cytoskeleton forms a contiguous network within all eukaryotic cells and is associated with the function of many cellular processes, such as cell division and development, cell polarity, and organelle movement (Kim et al., 2005; Sparkes et al., 2009; Yokota et al., 2009). During cell growth and development, as well as mechano-stimulation and in response to stress, the actin array undergoes rapid and highly regulated changes (Shimada et al., 2006; Hardham et al., 2007, 2008; Li et al., 2014). Plenty of studies have demonstrated that the actin cytoskeleton acts a pivotal part in plant basic defensive reaction and non-host resistance to various pathogens (Kobayashi et al., 1994; Hardham et al., 2007; Tian et al., 2009). Indeed, some of the earliest work by Kobayashi et al. (1994) indicated that cytoskeleton became radially arranged in mesophyll cells of flax around haustorium or at infection sites before penetration after infected with incompatible isolates of the *Melampsora lini*. More recently, Henty-Ridilla et al. (2013) mentioned the intrinsic quality and timing of induced changes to the actin microfilaments in *Arabidopsis* during the infection of pathogens. The early transient overexpress of actin resulting in increasing of actin filaments density and proved to be involved in pathogen-associated molecular pattern (PAMP)-triggered immunity (PTI) by using adapted and non-adapted microbes and treatments with microbial-associated molecular patterns (MAMPs). The microfilament cytoskeleton also participated in race-specific resistance; for example, previous work demonstrated that *AtADF4* mediates effector-triggered immunity (ETI) signaling through recognition of signaling activated in response to the recognition of the *Pseudomonas* effector AvrPphB (Tian et al., 2009).

The organization of the eukaryotic actin cytoskeleton is tightly regulated, and undergoes induced changes in response to a suite of internal and external stimuli. Indeed, a plethora of actin binding proteins (ABPs) have been demonstrated to regulate actin filament remodeling, among which include the actin-related protein 2/3 (Arp2/3) complex, profilin (PRF), actin depolymerizing factor (ADF), adenylate-associated protein (CAP), and Rac (a monomeric Rho-GTPase) (Hussey et al., 2006). A key step in this organization is actin nucleation, the key rate-limiting step necessary to ensure proper filaments formation (Campellone and Welch, 2010). Actin nucleation is regulated in large part by the activity of the Arp2/3 complex, with formins identified as the major actin nucleator (Chesarone and Goode, 2009). In most examples characterized to date, the ARP2/3 complex, consisting of two actin-like proteins (Arp2 and Arp3) and five unrelated subunits (Mullins et al., 1998). The remaining five subunits are commonly named according to their sizes, and in plants are referred to as ARPC1 (actin-related protein complex-1), ARPC2, ARPC3, ARPC4, and ARPC5 (Machesky et al., 1994). Although this complex has been studied more than 20 years (Machesky and Gould, 1999), a full understanding of its regulation in response to the perception of stress and developmental cues has remained elusive, and has been a topic of intense study. Genetic data indicates the Arp2/3 complex is essential in yeast (Winter et al., 1999). However, it remains unclear if each of the functional subunits is essential for the overall function of the Arp2/3 complex. For example, in *Saccharomyces cerevisiae*, deletion of the ARPC1 or ARPC2 caused lethality and severe reductions in viability. Similarly, the Arp2/3 complex in *Arabidopsis* has been revealed to control cell morphogenesis, while less is known about its role(s) in plant immunity (Li et al., 2003).

Stripe rust fungi are obligate biotrophic plant pathogens that would not grow without living hosts and cause damage worldwide (Tang and Chen, 2002). Wheat stripe rust, caused by *Puccinia striiformis* f. sp. *tritici* (*Pst*), occurs world-wide, and is one of the most destructive diseases of wheat in many cool and temperate regions (Wellings et al., 2003). Resistance of wheat cultivars against *Pst* is usually race specific and mediated by gene-for-gene resistance (Flor, 1971). Resistant cultivars are planted in large areas contribute to the disease control. Thus, it is crucial for understanding the mechanism of wheat against *Pst* and searching for new ways to control the wheat stripe rust disease. Plants defense fungal invasion through innate immune system called zigzag model (Jones and Dangl, 2006). PAMPs recognized as slowly evolving molecules, including non-pathogens, resulting in PTI. These include bursts of calcium and reactive oxygen species (ROS). Pathogens disrupt PTI by delivering effector molecules into their host during infection. In proper order, plants target these pathogen effectors via R proteins that activate ETI. ETI is pathogen strain- or race-specific and is associated with rapid host cell death, termed the hypersensitive response (HR), and leaded to systemic acquired resistance (SAR) in the host.

In the current study, we describe the characterization of the ARPC3 subunit of the wheat Arp2/3 complex. *TaARPC3* was identified as an evolutionarily conserved subunit, and data presented herein demonstrate a role for ARPC3 in regulation of actin cytoskeletal function during plant-pathogen interactions. Complementation of the *S. cerevisiae* mutant, *arc18*, with *TaARPC3* recovered developmental and actin-associated phenotypes, and in wheat, knock-down of *TaARPC3* reduced the resistance to *Pst* in an actin-dependent manner. Taken together, these results suggest that ARPs also play an important role in host response against fungal pathogens.

## Materials and Methods

### Plant Materials and Fungal Isolates

Wheat cultivar Suwon11 and *Pst* races CYR23 and CYR31 were used in this study. The method used for growing and inoculating of wheat seedlings were described by Kang and Li (1984). Suwon11 and CYR23 constituted an incompatible interaction that exhibited a typical HR, while Suwon11 was highly susceptible to CYR31 (Cao et al., 2003). CYR23 was maintained on wheat cv. Mingxian 169, whereas CYR31 maintained on wheat cv. Suwon 11.

To evaluate the expression levels of *TaARPC3* in response to *Pst* infection, wheat leaves were harvested at 0, 6, 12, 18, 24, 36, 48, 72, and 120 hour post-inoculation (hpi) based on previous microscopic observations of the interactions between wheat and *Pst* (Wang et al., 2007). To analyze the silencing efficiency of virus-induced gene silencing (VIGS) and the relative expression of *TaCAT1* and *TaSOD*, the fourth leaves of *TaARPC3*-knockdown wheat plants were sampled at 0, 24, 48, and 120 hpi following infection with *Pst.*

For hormone treatment, the 10-day-old wheat seedlings were sprayed with 500 μM salicylic acid (SA), 100 μM methyl jasmonate, 100 μM ethephon, 100 μM abscisic acid (ABA) and sampled at 0, 2, 6, 12, and 24 hpi. Wheat leaves inoculated with sterile distilled water were used as MOCK-inoculation controls.

### Quantitative RT-PCR Analysis (qRT-PCR)

Total RNA was isolated using the Biozol reagent (Invitrogen, Carlsbad, CA, United States). 3 μg of RNA aliquot of each sample was subjected to first strand cDNA synthesis using the oligo (dT)^18^ primer and Promega Reverse Transcription System (Promega, Madison, WI, United States). The primers (Supplementary Table S3) were designed according to the procedures as described by Livak and Schmittgen (2001) and quantization Real-Time PCR (qRT-PCR) assays were performed on an ABI-7500 system (Applied Biosystems, Foster City, CA, United States). The transcript level of *TaARPC3* was quantified using the comparative 2^-ΔΔCT^ method (Livak and Schmittgen, 2001) with the wheat *Elongation factor 1* (*TaEF-1α*) used as control (GenBank accession number Q03033) (Supplementary Table S3). Transcript abundance of each reaction was assessed in triplicate.

### Identification and Sequence Analysis of TaARPC3

*Arabidopsis thaliana* actin related protein complex subunit 3 ARPC3 (Li et al., 2003; Cvrčková et al., 2004) was used to screen (*in silico*) the EST databases of wheat constructed in our lab (Ma et al., 2009; Wang et al., 2009, 2010). A 1091-bp unisequence (Genbank accession number AK336075.1) exhibiting high homology to the *A. thaliana* homolog, *AtARPC3*, was obtained from the cDNA library (Yu et al., 2010). Based on this sequence, we used specific primers TaARPC3-F and TaARPC3-R (Supplementary Table S3) to amplify the sequence of *TaARPC3*. The cloned open reading frame (ORF) was constructed into pGEM-T Easy (Promega, United States) for sequencing.

The cDNA sequence of *TaARPC3* was analyzed *in silico* using the online BLAST^1^ and the ORF Finder software^2^ in NCBI. The amino acid sequence of TaARPC3 was analyzed with Pfam^3^ and ScanProsite^4^ for conserved domain identification. Multiple sequence alignments were performed using CLUSTALX2.0 (Chenna et al., 2003) and DNAMAN6.0 (Lynnon BioSoft, United States). A phylogenetic tree based on the neighbor-joining method was performed with the Mega 6 software (Tamura et al., 2007).

### Yeast Mutant Complementation Assay

The *S. cerevisiae* diploid mutant strain Y06714 (MATa; ura3Δ0; leu2Δ0; his3Δ1; met15Δ0; YLR370c::kanMX4) and the wild type strain (WT) BY4741 (MATa; his3Δ1; leu2Δ0; met15Δ0; ura3Δ0) were obtained from EUROSCARF collection. The complete ORF of *TaARPC3* was cloned into pDR195. The transformed cell (Δ*arc18*+TaARPC3) was obtained by transforming the reconstructed vector (pDR195-*TaARPC3*) into the mutant strain Y06714. The transformed cell with empty vector pDR195 (Δ*arc18*+empty) was used as control. Transformants were selected on SC-U medium at 30°C and validated using PCR.

To investigate the yeast cell survival under stress, the transformed cells with pDR195 (Δ*arc18*+empty) and pDR195-*TaARPC3* (Δ*arc18*+ TaARPC3) and wild yeast cells (WT BY4741) were cultured on SC medium with 0.1mM H_2_O_2_, 2 M Sorbitol, 0.3 M NaCl and 0.1 M CaCl_2_, respectively. Transformed cells with a starting optical density at 600 nm (OD_600_) of 0.2 were cultured in yeast medium. The fluorescent dye 4′, 6-diamino-2-phenylindole (DAPI) was added to the cell suspensions at a concentration of 5 μg ml^-1^ to counter-stain nuclei. The cells were stained with 0.5 μM TRITC-phalloidin to observe F-actin structures (Bereiterhahn and Kajstura, 1988). Cells were incubated at 30°C in phosphate buffer saline (PBS) for 30 min without shaking, followed by washing with sterile distilled water, then resuspended in distilled water. Cells were examined by fluorescence microscopy using an Olympus BX-53 microscope (Olympus, Corp., Tokyo) under blue light excitation by epifluorescence microscopy (excitation filter, 485 nm; dichroic mirror, 510 nm; and barrier filter, 520 nm).

### Subcellular Localization of TaARPC3:GFP Fusion Protein in Wheat Protoplasts

The full-length cDNA of *TaARPC3* was cloned into the pCaMV35S::GFP vector via *Xba*I*/Bam*HI digestion and cloning. Protoplast isolation in wheat leaves was performed as previously described (Graham, 2002). The recombinant plasmid pCaMV35S:TaARPC3:GFP, empty vector pCaMV35S:GFP were transformed into wheat protoplasts by PEG4000. The transformed wheat mesophyll protoplasts were incubated in a dark chamber plates at 24°C for 12–36 h. GFP fluorescence was detected with an Olympus FV1000 confocal laser microscope equipped with a 488 nm filter (Olympus, Tokyo, Japan).

### BSMV-Mediate Silencing of *TaARPC3* in Wheat cv. Suwon 11

For VIGS, a 214-bp fragment of *TaARPC3* with *Not*I and *Pac*I (Supplementary Table S3) was derived from its coding sequence and amplified by RT-PCR to construct the BSMV:γ plasmid for gene silencing (Holzberg et al., 2002). To ensure the specificity for target gene silencing, multi-alignment and BLAST results indicated that the insert sequence of *TaARPC3* was located at the non-conservative region and without off target possibility (**Supplementary Figure S1** and Table S2). The BSMV RNAs were obtained *in vitro* from linearized plasmids γ-*TaPDS*, γ-*TaARPC3*, γ, α, β (Petty and Jackson, 1990) using the Message T7 transcription kit (Ambion, Austin, TX, United States) according to the manufacturer’s instruction. A 7.5 μl portion of each three transcripts of BSMV viruses (wild type or target gene carrying types) were mixed with 45 μl FES and directly applied by gently sliding the pinched fingers from the base to the tip five times with a gloved finger (Scofield et al., 2005; Bruun-Rasmussen et al., 2007). After inoculation with BSMV at the second leaf stage, plants were placed in the dark chamber for 24 h, and subsequently transferred to a growth chamber at 25 ± 2°C with a 16 h photoperiod and examined for symptoms. In all experiments, the recombinant virus BSMV:*TaPDS* was used as a positive control, negative control were inoculated with BSMV: γ. When the photo-bleaching phenotype (indicating the *PDS* gene silence) was observed, the fourth leaves of *TaARPC3* silencing group were inoculated with urediospores of *Pst* race CYR23 and CYR31, respectively. The fourth leaves were collected at 24, 48, and 120 hpi for histological observation and qRT-PCR. The resistance or susceptible phenotypes were visible at 14 dpi.

### Fungal Biomass Analyses and Quantification of Disease Severity

Disease severity was assessed by counting the number of uredinia within standardized leaf area sections following infection with *Pst.* Leaves was collected randomly to avoid bias. The results were exhibited in the mean values of the number of uredinia on knock-down plants compare to the controls. Fungal biomass was analyzed by qRT-PCR as described previously (Livak and Schmittgen, 2001; Cheng et al., 2016). Genomic DNA were performed using the CTAB method (Del et al., 1989) from samples collected at 120 hpi after infected with *Pst*. Standard curve was generated by the plasmid carried the fragment of *PsEF1* and *TaEF1* as described (Liu J. et al., 2016; Liu P. et al., 2016) (Supplementary Table S3).

### Histological Observation of Host Response and Pathogen Growth

The host response and *Pst* growth in *TaARPC3* know-down plants were observed by light microscopy. Stained leaf segments were fixed and cleared in ethanol/acetic acid (1:1 v/v). Auto-fluorescence of infected mesophyll cells was observed as a necrotic death area with an Olympus BX-51 microscope (Olympus Corp., Tokyo) (excitation filter, 485 nm; dichroic mirror, 510 nm; and barrier filter, 520 nm). Infection sites and lengths of infection hyphae were measured under the blue light excitation. H_2_O_2_ accumulation around the infection site was detected by using 3,3-diaminobenzidine (DAB; Amresco, Solon, OH, United States) staining, observed by the differential interference contrast optics. Fifty infection sites were randomly selected for each leaf segment, per treatment. Methods were taken as described previously (Wang et al., 2007), including identification of successful infection sites, hyphae staining and measurement of fungal structures.

### Statistical Analyses

Standard errors of deviation were calculated using Microsoft Excel. Statistical significance was assessed by a Student’s *t*-test (*P* < 0.05) using SPSS software (SPSS, Inc., Chicago, IL, United States).

## Results

### TaARPC3 Encodes a Typical Component of Arp2/3 Complex

**FIGURE 1:**
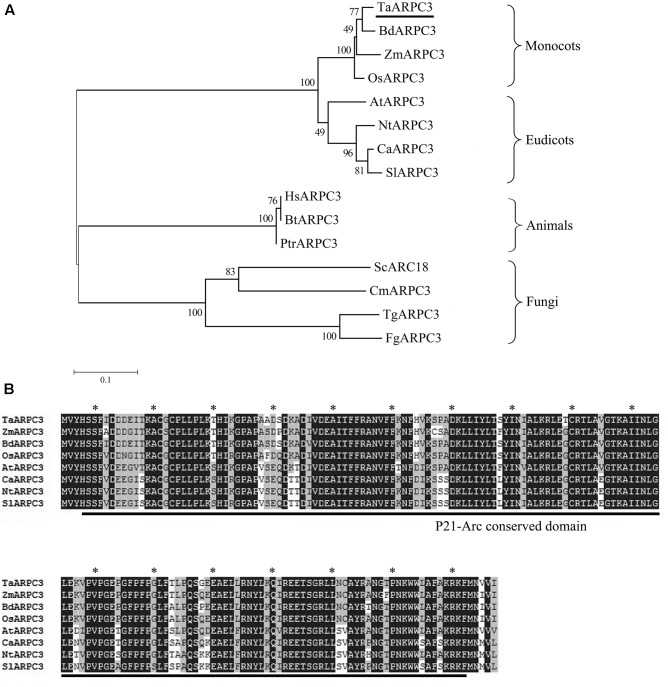
**(A)** Phylogenetic analysis of *ARPC3* genes. A phylogenetic analysis of ARPC3 in *Triticum aestivum* (TaARPC3, KU746328), *B. distachyon* (BdARPC3, XP_003571891), *Oryza sativa* (OsARPC3, XP_006647107), *Arabidopsis thaliana* (AtARPC3, NP_564757), *Zea mays* (ZmARPC3, NP_001136734), *Nicotiana tabacum* (NtARPC3, XP_016458972.1), *Capsicum annuum* (CaARPC3, XP_016554076.1), *Solanum lycopersicum* (SlARPC3, XP_004242738.1), *Human species* (HsARPC3, NP_001265485), *Bos taurus* (BtARPC3, NP_001029443.1), *Pan troglodytes* (PtrARPC3, NP_001238842.1), *Saccharomyces cerevisiae* (ScARPC3, NP_013474.3), *Candida maltose* (CmARPC3, EMG45739.1), *Trichoderma guizhouense* (TgARPC3, OPB39669.1), and *Fusarium graminearum* (FgARPC3, KIL85740.1). **(B)** Multiple amino acid sequences alignment of TaARPC3 with AtARPC3, BdARPC3, OsARPC3, and TuARPC3. Amino acid identity (black boxes) and similarity (gray boxes) are shown within the protein kinase domain and the most highly conserved residues are highlighted with asterisk.

A wheat 526-bp homolog of actin related protein was isolated from wheat cv. Suwon 11 by homology-based cloning and the obtained cDNA was designated as *TaARPC3*. Blast analysis of *TaARPC3* nucleotide sequence in the *Triticum aestivum* cv. Chinese spring (CS) genome sequence revealed that at least three copies were present, localized on chromosome 7A, 7B, and 7D (**Supplementary Figure S1A**). The *TaARPC3* exhibited 97.84, 92.78, and 97.97% identities with *TaARPC3-7A, TaARPC3-7B, TaARPC3-7D*, respectively. The predicted ORF of *TaARPC3* encodes a protein of 174 amino acid with a hypothetical molecular weight of approximately 21 kDa and an isoelectric point (PI) of 6.71. A phylogenetic analysis of TaARPC3 with BdARPC3, OsARPC3, AtARPC3, ZmARPC3, NtARPC3, CaARPC3, SlARPC3, HsARPC3, BtARPC3, PtrARPC3, MScARPC3, CmARPC3, TgARPC3, and FgARPC3 resulted in that TaARPC3 clusters with ZmARPC3, BdARPC3 and OsARPC3, all of which are members of actin related proteins in monocotyledons (**Figure 1A**). Nucleic acid sequence analysis revealed that *TaARPC3* shared 91% identity with *BdARPC3* from *Brachypodium distachyon*. Multiple amino acid sequences alignment of TaARPC3 with BdARPC3, OsARPC3, AtARPC3, ZmARPC3, NtARPC3, CaARPC3, and SlARPC3 showed that *TaARPC3* is predicted to encode proteins with the unique conserved domains of ARP2/3 complex ARPC3 (**Figure 1B**). These results indicate that *TaARPC3* (Genbank accession number KU746328) is a typical member of the Arp2/3 complex in wheat.

### *TaARPC3* Can Partially Rescue the *arc18* Mutant in *Saccharomyces cerevisiae*

**FIGURE 2:**
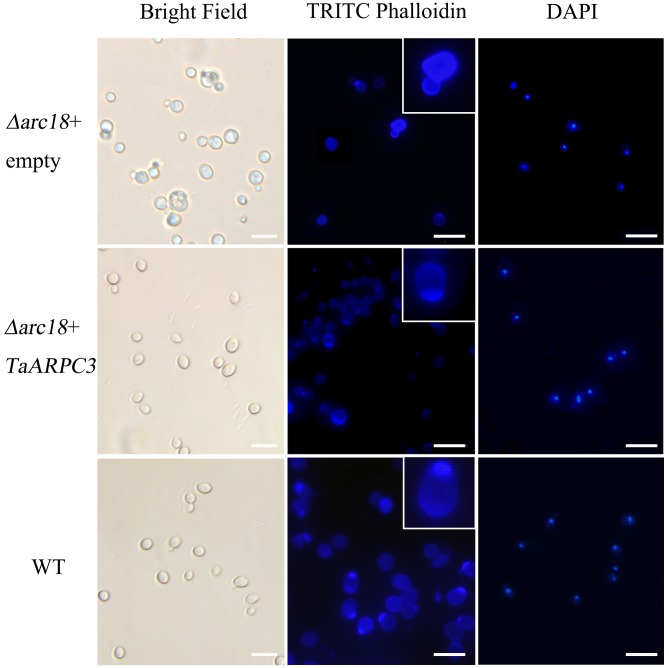
Complementation of *TaARPC3* in yeast recovers the loss of actin morphology. Cell morphology observed using brightfield microscopy. Cells were stained with TRITC-phalloidin and DAPI. Enlarged views indicate the accumulation of F-actin. Bar, 10 μm.

A recent study demonstrated a role for the Arp2/3 complex in the actin-associated control of cell division and polarity in *S. cerevisiae* (Cabrera et al., 2011). *TaARPC3* shared 38% similarity with *arc18* (aka ARPC3) from *S. cerevisiae* (**Supplementary Figure S1B**). To evaluate the function of *TaARPC3*, we tested the ability of *TaARPC3* to complement the *arc18* mutation in yeast. To do this, the *TaARPC3* coding sequence into pDR195 vector was transformed into the *S. cerevisiae* mutant strain Y06714 and heterologously expressed. As shown in **Figure 2**, *S. cerevisiae* cells harboring the empty vector pDR195, as well as the wild-type (untransformed) strain, BY4741 (control), showed a similar growth phenotype (i.e., rapid growth on SC medium than the mutant strain). The *S. cerevisiae* mutant Y06714 (aka, *Δarc18*) exhibited several morphological defects. As shown, mutant cells vary in size and shape when compared to the wild-type and the complemented strain. Cells were stained with TRITC-phalloidin to assess the subcellular organization of the F-actin, which facilitates observation of F-actin structures. As shown in **Figure 2**, staining revealed F-actin aggregation in the budding site in the complemented strain, as well as accumulation of actin dots at the septum-forming position. In the mutant strain, actin was observed as concentrated foci at the cell ends, adjacent to the developing septum. No significant difference was observed by fluorescence microscopy of DAPI-stained cells. In total, these data reveal a loss of actin cables and depolarized actin patches, suggesting that *Ta*ARPC3 may play a role in maintaining the polarized distribution of actin patches within the cell.

**FIGURE 3:**
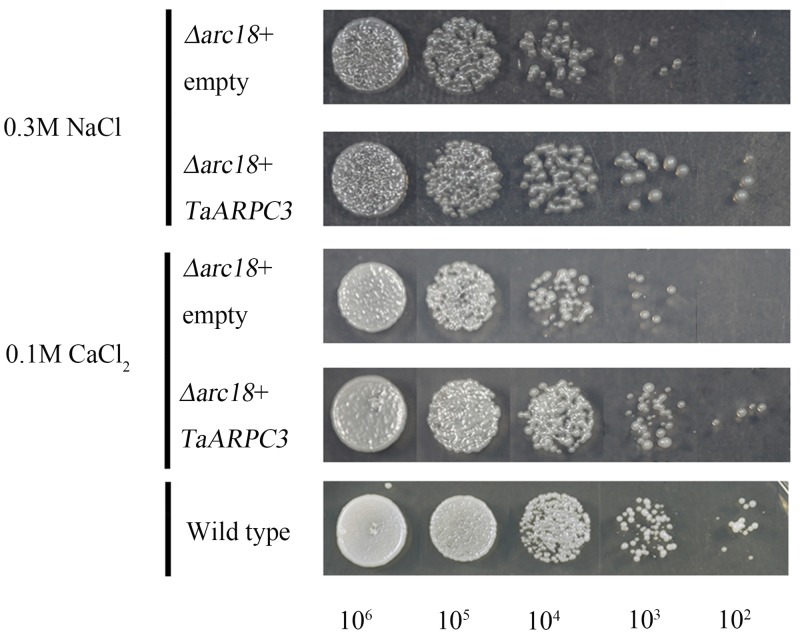
Effects of expression of *TaARPC3* in yeast cells. Survival of the mutant yeast cells Δ*arc18* expressing empty pDR195 vector (Δ*arc18*+empty) or pDR195-TaARPC3 (Δ*arc18*+TaARPC3) were spotted on solid medium in combination with different stress treatment. 0.3 M NaCl, solid medium containing 0.3 M NaCl; 0.1 M CaCl_2_, solid medium containing 0.1 M CaCl_2_. The final densities were 10^6^, 10^5^, 10^4^, 10^3^, and 10^2^ (cell/ml) following dilution with sterile water.

The Arp2/3 complex has been demonstrated to be required for cellular response to various abiotic stresses. To determine if *TaARPC3* could complement the *S. cerevisiae* mutant phenotypes with respect to the perception of cell stress, we tested the function of *TaARPC3* in *S. cerevisiae* under a variety of cellular stresses. As shown in **Figure 3**, we observed a significant increase in the growth rate of the complemented strain Y06714 compared with the *S. cerevisiae arpc3* mutant carrying the empty expression vector pDR195. In SC-U media containing 0.1 mM H_2_O_2_ (oxidative stress), 2 M sorbitol (osmotic stress), 0.3 M NaCl (salt stress), and 0.1 M CaCl_2_, similar results were observed (**Figure 3** and **Supplementary Figure S2**). Yeast cells expressing pDR195 and pDR195-*TaARPC3* grew more slowly than WT cells on stress-inducing medium, while the complemented strain containing pDR195-TaARPC3 grew at near wild-type levels (**Figure 3** and **Supplementary Figure S2**), indicating complementation by *TaARPC3.* Taken together, these results indicate that the *TaARPC3* participates in abiotic stress signaling.

### TaARPC3:GFP Fusion Protein Was Found in the Nucleus and Cytoplasm in Wheat Protoplast

**FIGURE 4:**
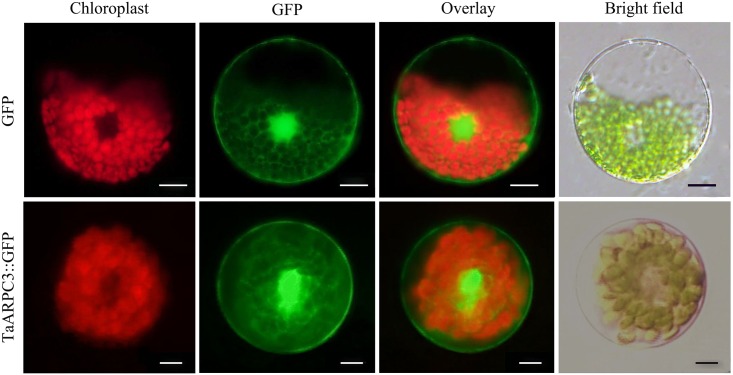
Subcellular localization of the TaARPC3 protein. Expression of TaARPC3-GFP fusion protein and only GFP (control) in wheat seedlings protoplasts. The green channel shows the localization of GFP and TaARPC3-GFP. Bar, 20 μm.

To determine the subcellular localization of TaARPC3 in wheat, the green fluorescent protein (GFP) gene was fused with *TaARPC3*, and then expressed in wheat protoplasts. As showed in **Figure 4**, empty pCaMV35S:GFP vector, as a control, revealed a diffuse, non-specific cellular address, while cells expressing GFP-tagged TaARPC3 was localized predominantly in the nucleus and cytoplasm.

### *TaARPC3* Is Differentially Induced by *Pst* Infection and Hormone Treatment

**FIGURE 5:**
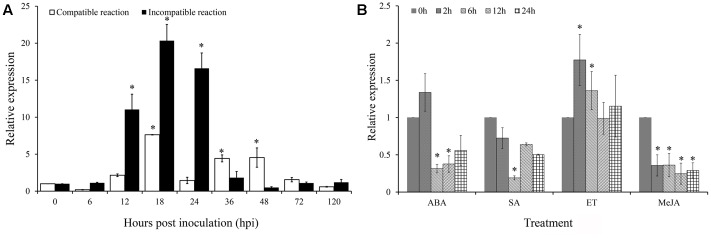
Quantitative Real-Time PCR expression analyze of *TaARPC3* in wheat leaves. **(A)** The expression level of *TaARPC3* in wheat leaves was induced in incompatible reaction between wheat-*Pst* interaction. **(B)** The transcription of *TaARPC3* in wheat response to ET, ABA, MeJA, and SA treatment. Error bars represent the variations among three independent replicates. The data were normalized to wheat the *TaEF-1a* gene. Wheat leaves treated with distilled water were included as a control. Asterisks indicate significant difference (*P* < 0.05) from 0 hour after inoculation using Student’s *t*-test.

To determine the expression profiles of *TaARPC3* mRNA, we next monitored the expression of *TaARPC3* during the interaction between wheat and *Pst* using qRT-PCR. As shown in **Figure 5A**, during an incompatible interaction, *TaARPC3* transcripts were up-regulated as early as 12 hpi, with peak expression at 18 hpi; transcript levels gradually reduced to pre-inoculation levels at 36–120 hpi. During a compatible interaction, *TaARPC3* was also induced from the 12 hpi to 48 hpi; however, the transcript levels of *TaARPC3* were much higher during an incompatible interaction than that during a compatible interaction at 12–24 hpi. These results support our hypothesis that *TaARPC3* is likely to be associated with resistance of wheat against *Pst* infection.

To extend these assays, we next evaluated the expression of *TaARPC3* as a function of hormone perception and signaling. To do this, wheat seedlings were treated with ethylene (ET), ABA, SA, and jasmonic acid (JA) to evaluate the possible linkages between immune signaling, susceptibility, and hormone biosynthesis. As shown in **Figure 5B**, when seedlings were treated with ET, the expression level of *TaARPC3* was significantly induced at 12 hpi post-treatment (hpt), with an approximate 2.5-fold change in expression, after which time, transcript levels returned to levels similar to control plants (**Figure 5B**). After treatment with ABA, *TaARPC3* transcript levels were slightly induced at up to 2 hpi, and after 2 hpi, were down-regulated (**Figure 5B**). Treatment with SA and MeJA was observed to down-regulate *TaARPC3* expression.

### Silencing of *TaARPC3* Results in a Decrease in Resistance against *Pst*

**FIGURE 6:**
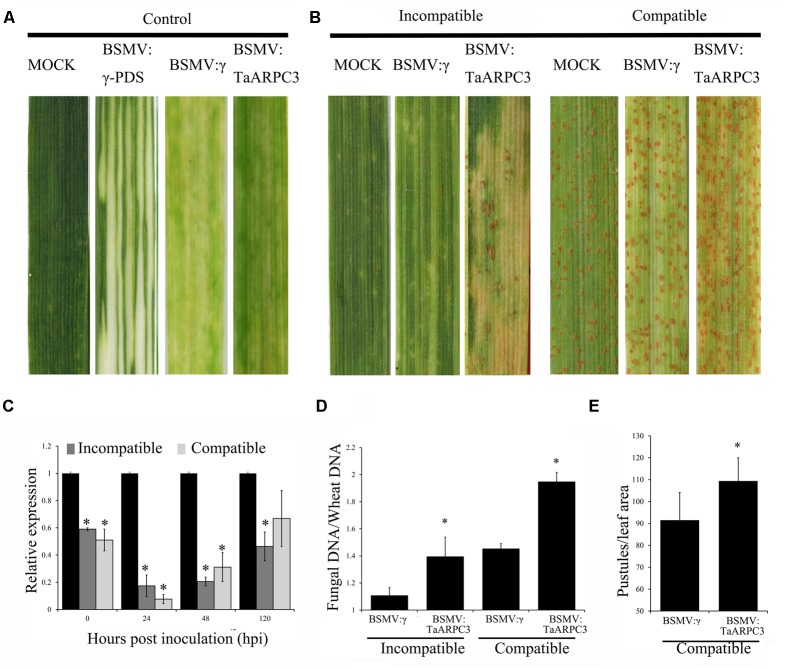
The instantaneous silencing of *TaARPC3* by BSMV-VIGS method. **(A)** Wheat leaves treated with 1× Fes buffer (MOCK) shown no phenotypic changes. Mild chlorotic mosaic symptoms were detected on the plants inoculated with BSMV:γ, BSMV:*PDS* or BSMV:*TaARPC3*. **(B)** Phenotypes of the fourth leaves infected with urediospores of *Pst* CYR23 (avirulent) or CYR31 (virulent). Representative leaves were photographed at 15 dpi. **(C)** Relative expression levels of *TaARPC3* in knockdown plants challenged with CYR23 or CYR31 at 0, 24, 48, and 120 dpi by qRT-PCR assay. The relative transcript level of *TaARPC3* was calculated by the comparative threshold method (2^-ΔΔCT^). Error bars represent the standard deviations among three independent replicates. Asterisks indicate significant difference (*P* < 0.05) in relative expression as compared to mock inoculated samples. **(D)** Fungal biomass analysis assay in *TaARPC3*-knowdown plants after inoculation CYR23. Asterisks indicate significant difference (*P* < 0.05) in relative expression as compared to BSMV:inoculated. **(E)** Quantification of the uredinial density in the *TaARPC3*-knowdown plants after inoculation CYR31. Asterisks indicate significant difference (*P* < 0.05) in relative expression as compared to BSMV:inoculated.

The VIGS system is a rapid and effective reverse-genetic approach to characterize gene function in wheat (Yin et al., 2011). To functional the *TaARPC3* in wheat-*Pst* interaction, we knocked down *TaARPC3* using the VIGS and analyzed the impact of this on wheat response to pathogen infection. As exhibited in **Figure 6A**, 10 days after BSMV inoculation, mild chlorotic mosaic phenotypes were observed on the fourth leaves in BSMV-inoculated plants, with leaves inoculated BSMV:*γ-PDS* displaying obvious signs of photobleaching (**Figure 6A**); this indicates effective gene silencing. Next, the fourth leaves of wheat with BSMV-inoculated were inoculated with the avirulent race (CYR23) and virulent race (CYR31) to Suwon 11, respectively (**Figure 6B**). After 15 days, CYR23 elicited a pronounced HR on leaves inoculated with BSMV:γ, BSMV:*TaARPC3*-inocuated leaves, with uredia production observed on BSMV:*TaARPC3*-inocuated leaves (**Figure 6B**). When leaves were challenged with *Pst* CYR31, leaves inoculated with BSMV:*TaARPC3* show more uredia produced than observed in mock plants and those inoculated with BSMV:γ (**Figure 6B**). To confirm silencing efficiency, the transcript level of *TaARPC3* in *TaARPC3* knock-down plants were evaluated by qRT-PCR. We observed that the relative expression at each time point was significantly suppressed as much as 93% (**Figure 6C**). Next, to quantify the disease severity in the silenced plants, fungal biomass was found to be significantly increased (ca. 1.34-fold increased compared to control infected with CYR23; **Figure 6D**). The same result was observed after inoculated with CYR31. Quantification of the expansion in uredinia development in leaves was observed to increased 1.19-fold compared with controls treated with BSMV::γ alone (**Figure 6E**). These results indicate that *TaARPC3* is required for defense signaling in wheat during *Pst* infection.

### Host Response and *Pst* Growth in Knock-Down Plants

**FIGURE 7:**
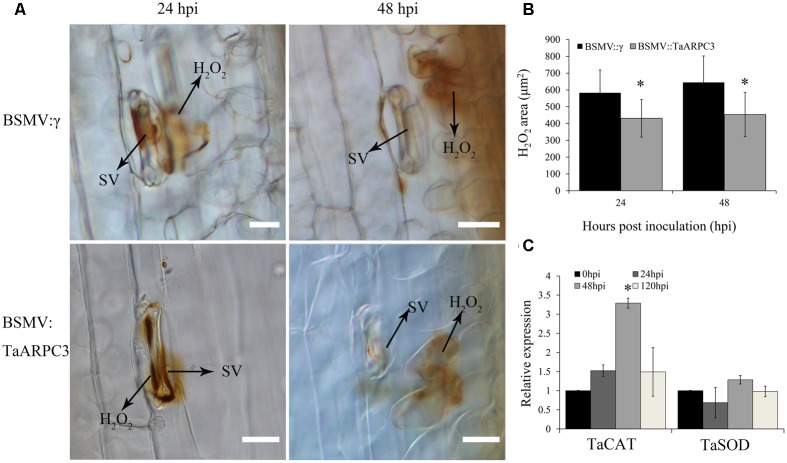
H_2_O_2_ accumulation observations of know-down plants that inoculated with incompatibility race CYR23. **(A)** H_2_O_2_ accumulation was measured at infection sites by microscopy. HMC, haustorial mother cell; IH, infection hypha; HR, hypersensitive response; SV, sub-stomatal vesicle. Bar, 20 μm. **(B)** The area of H_2_O_2_ staining by 3,3′-diaminobenzidine (DAB) was measured by DP-BSW software in *TaARPC3* knockdown plants at 24 and 48 hpi after inoculation. **(C)** Relative expression of *TaCAT* and *TaSOD* in *TaARPC3* knockdown plants. Error bars represent the variations among three independent replicates. Asterisks indicate significant difference (*P* < 0.05) from BSMV:γ using Student’s *t*-test.

To further understand the how *TaARPC3* participates in wheat resistance, H_2_O_2_ accumulation was evaluated. As shown in **Figure 7**, H_2_O_2_ production in *TaARPC3*-silenced wheat showed a significant reduction compared with BSMV:γ-treated plants after CYR23 inoculation (**Figures 7A,B**). To confirm a decrease in ROS accumulation, we next assayed the expression levels of selected genes involved in the ROS signaling pathway [e.g., catalase (*TaCAT*) and superoxide dismutase (*TaSOD*)] in *TaARPC3-*knockdown plants following infection with *Pst*. As shown in **Figure 7C**, we observed an increase in the accumulation of *TaCAT* in *TaARPC3*-knockdown plants compared with mock-inoculated or BSMV:γ control plants. Conversely, *TaSOD* transcripts showed no significant change after *TaARPC3* silencing.

**FIGURE 8:**
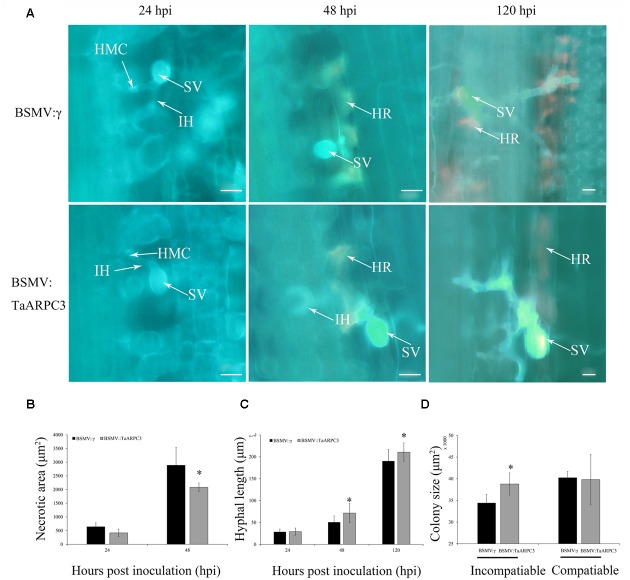
Histological observations of necrotic cell death in wheat leaves treated with BSMV and infected with incompatibility race CYR23. **(A)** Necrotic cell death was observed by epifluorescence at 24, 48, and 120 hpi. HMC, haustorial mother cell; IH, infection hypha; HR, hypersensitive response; SV, sub-stomatal vesicle. Bar, 20 μm. **(B)** Necrotic cell death was quantified as the area of auto-fluorescence. **(C)** Hyphal lengths were quantified from the junction of the sub-stomatal vesicle to the hyphal tip. **(D)** Colony size was measured as the area of auto-fluorescence. Asterisks indicate a significant differences (*P* < 0.05) from BSMV:γ using the Student’s *t*-test.

In addition to the induction of H_2_O_2_ accumulation, we also observed that pathogen-induced cell death exhibited a similar decrease in the *TaARPC3*-knowdown plants (**Figure 8A**), as evaluated by microscopy (**Figure 8B**). A histological examination of *Pst* growth was performed in both control and silenced plants by analyzing the length of hyphae and the number of hyphal branches and the formation of haustorial mother cells at infection sites (Supplementary Table S1). In short, we found that the hyphal branch between the BSMV:*TaARPC3* and BSMV:γ plants at 24 hpi showed no significant change after inoculation with both CYR23 and CYR 31 (Supplementary Table S1), while the hyphal length was longer in *TaARPC3* knock-down plants (**Figure 8C** and Supplementary Table S1). In addition, the colony area per infection site in the incompatible interaction was obviously larger in *TaARPC3* silenced plants compare with the control group at 120 hpi in incompatible interaction (**Figure 8D** and Supplementary Table S1). In total, these results indicated that a reduction in *TaARPC3* expression compromises resistance signaling in response to *Pst* infection.

## Discussion

In the current study, we isolated the wheat *TaARPC3* gene, with duplication across the chromosomes 7A, 7B, and 7D, which has a similar conserved P21-Arc domain to that found in numerous other ARPC3 proteins from a wide variety of monocotyledonous plants. As a function of plant defense signaling, our data extend our current understanding in this area and provide a foundation from which to extend studies both in wheat, as well as in numerous other plants, to define the role of this complex during immune signaling and the control of actin cytoskeletal organization during host infection by pathogens.

Plant cells responding to fungal attack undergo numerous changes within a multitude of cellular processes. Among these, rearrangements of the cytoskeleton have been demonstrated to play a major role in the progress of fungal penetration and host resistance. For example, the actin cytoskeleton has been demonstrated to act as a key downstream effector of external and internal stimuli during a wide range of biological phenomena in plants (Vantard and Blanchoin, 2002). Indeed, it is widely accepted that Arp2/3 functions as a regulator of actin filament dynamics by polymerizing the branched F-actin and promoting actin nucleation of actin filament (Higgs and Pollard, 2001; May, 2001; Welch and Mullins, 2002). However, its role in fungal defense is less defined. In this study, we analyzed the function of *TaARPC3* in wheat against *Pst*. Numerous studies have shown that the actin cytoskeleton is associated with plant disease resistance (Tian et al., 2009; Porter et al., 2012; Fu et al., 2014; Tang et al., 2014; Zhang et al., 2016). As highlighted herein, our current work demonstrates that *TaARPC3* is required for resistance signaling in wheat following *Pst* infection. As a biotrophic parasite, *Pst* is depending on haustoria to absorb nutrition from the host wheat (Kang et al., 2002). The substomatal vesicle within the stomatal cavity as a symbol of an infection site was formed at 6–8 hpi, the primary infection produce hypha, haustorial mother cell, and haustorium initial at 12–18 hpi. From 24 to 144 hpi, hyphae branch and haustoria formed greatly and developed into mycelium. Indeed, and as a function of the specific regulation of resistance at distinct stages of infection, we observed that the expression of *TaARPC3* in an incompatible interaction was induced during the initial stages of infection; while in the compatible interaction, we observed only a slight delay in this response. At present, we do not know whether *TaARPC3* patriciates in either ETI, PTI, or both layers of immunity. Moreover, the function of this complex, both as a regulator of cortical actin function and as a downstream regulator (e.g., nuclear) of cytoskeletal function remains to be fully defined.

*TaARPC3* was found predominantly localized within the nucleus and cytoplasm in wheat protoplasts. In the budding yeast, *arc18* is recruited to the mitochondria and the *arc18* deletion mutant shows impaired mitochondrial transport (Fehrenbacher et al., 2005). While in most of higher plants, ARP2/3 coats the surface of biochemically and morphologically distinct organelles, in *Arabidopsis*, AtARP3 subunit is present in nuclei, the mitochondria, and chloroplasts (Zhang et al., 2013). Within these subcellular sites, AtARPC4 was found to be associated with microsomes and the nucleus (Kotchoni et al., 2009; Zhang et al., 2013), while AtARPC2 was found bound directly to micro-tubulins, and interacted with both actin filaments and microtubules (Havelková et al., 2015). To explain these differences, we hypothesize that one function of the Arp2/3 complex may be to regulate organelle positioning and the patterns of organelle transport as a distinct function of each of the different subunits. As a function of the work presented herein, we posit that *TaARPC3* may participate in resistance signaling in wheat through regulated changes in nuclear actin and/or organelle positioning.

In addition to nuclear- and organizational-specific changes in the cell, numerous studies have demonstrated a link between Arp2/3, actin, and second messenger perception pathways, including those triggered by hormones, Ca^2+^, and cAMP (Eun et al., 2001; Hepler et al., 2001; Moutinho et al., 2001; Friml et al., 2002). Herein, we observed an induction of *TaARPC3* upon exogenous ET application, and conversely, a reduction in *TaARPC3* expression following MeJA treatment, suggesting that *TaARPC3* may be an effector associated with ET and JA signaling pathways. Taken together with our observation of an enhancement in yeast cell response to environmental stress stimuli and the induced expression pattern of *TaARPC3* under avirulent *Pst*, it is reasonable to hypothesize that the Arp2/3 complex functions at the nexus of biotic and abiotic stress pathways. Coupled with our demonstration of its role in actin cytoskeletal organization, our data support this proposed role for Arp2/3, linking cell stress, signaling, and the organization of the actin cytoskeleton.

The abiotic and biotic stresses that induce an oxidative burst response has been shown to likely function as a protective mechanism during pathogen infection and environmental stress (Apel and Hirt, 2004; Bhattacharjee, 2005). In plants, the ROS burst, one of the earliest signal events, happens during the early stages of plant–pathogen interactions (Garcia-Brugger et al., 2006). As reported by Wang et al. (2007), the ROS bursts were detected at 12–24 hpi, in the wheat-*Pst* incompatible interaction. As demonstrated herein, the time point that *TaARPC3* was highly induced correlated with the induction of the ROS burst, a processes that is associated with both abiotic and biotic stress signaling. Further, in *TaARPC3*-silenced plants, the expression levels of *TaCAT* were also induced. Numerous reports have revealed that changes in cytoskeletal organization can lead to reactive oxygen bursts and subsequent cell death (Gourlay and Ayscough, 2005). For example, in yeast, a reduction in actin dynamics was shown to lead to a loss of mitochondrial membrane potential and elevated ROS levels (Gourlay et al., 2004). Similarly, overexpression of the phosphodiesterase *PDE2* rescues actin dynamics was shown to reduce oxidative stress sensitivity (Gourlay and Ayscough, 2006). As a possible mechanism for this response, cells bearing mutations in *ARP2* or *arc15* genes show decreased velocities of mitochondrial movement and a concomitant loss of all directed movement in mitochondrial morphology (Boldogh et al., 2001). Thus, actin cytoskeletal organization likely functions as a physiological regulator of ROS release from mitochondria and as a key element in the upstream activation of cell death pathways (Breitenbach et al., 2006).

In summary, our study demonstrates that *TaARPC3* likely participates in the regulation of actin cytoskeleton, and moreover, plays a key role in the host response to pathogen infection. In support of this hypothesis, our data show that *TaARPC3* was able to contribute to the stable of cytoskeleton to enhance wheat resistance via the management of ROS accumulation and cell death during the incompatible interaction of wheat and *Pst*.

## Author Contributions

JG and QM designed the experiment. TQ, JW, and QS performed the experiments and analyzed the data. TQ, JW, JG, BD, and QM wrote the manuscript.

## Conflict of Interest Statement

The authors declare that the research was conducted in the absence of any commercial or financial relationships that could be construed as a potential conflict of interest.

## Footnotes

1 https://blast.ncbi.nlm.nih.gov/Blast.cgi

2 https://www.ncbi.nlm.nih.gov/orffinder/

3 http://pfam.sanger.ac.uk/

4 http://prosite.expasy.org/scanprosite/

## Supplementary Material

FIGURE S1 **(A)** Aliment and VIGS site of *TaARPC3* of three copies of *TaARPC3* located on chromosomes 7A, 7B, and 7D. **(B)** Aliment of TaARPC3 with ARC18 in yeast.

FIGURE S2 Effects of expression of *TaARPC3* in yeast cells. 0.1 mM H_2_O_2_, solid medium containing 0.1 mM H_2_O_2_; 2 M Sorbitol, solid medium containing 2 M Sorbitol.
