# Supplementary figures and images for: TaARPC3, Contributes to Wheat Resistance against the Stripe Rust Fungus
Source: Front Plant Sci. 2017 Jul 18;8:1245. doi: 10.3389/fpls.2017.01245 (PMC5513970; doi:10.3389/fpls.2017.01245)

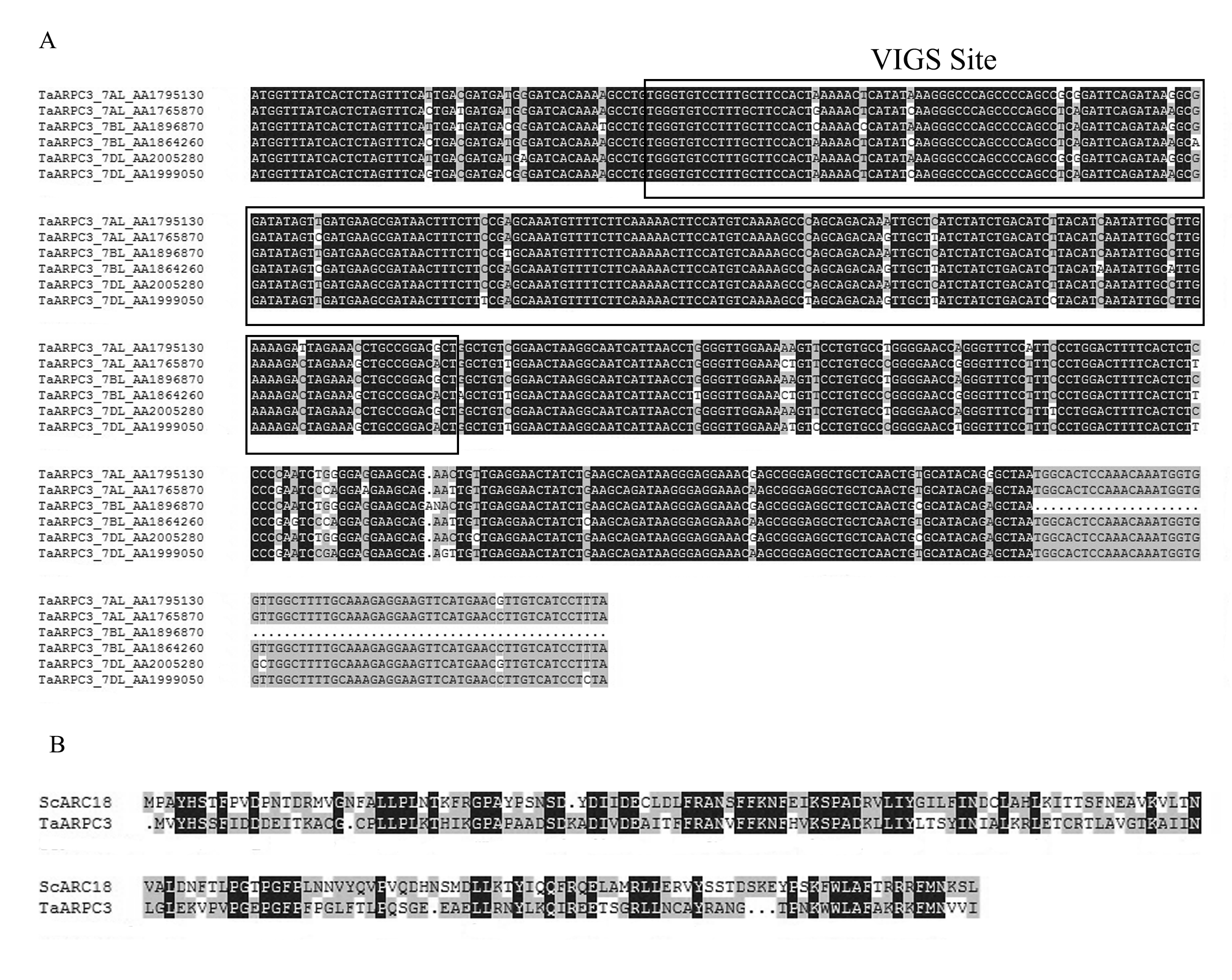

Supplement: FIGURE S1 — (A) Aliment and VIGS site of TaARPC3 of three copies of TaARPC3 located on chromosomes 7A, 7B, and 7D. (B) Aliment of TaARPC3 with ARC18 in yeast. [file Image_1.TIF]

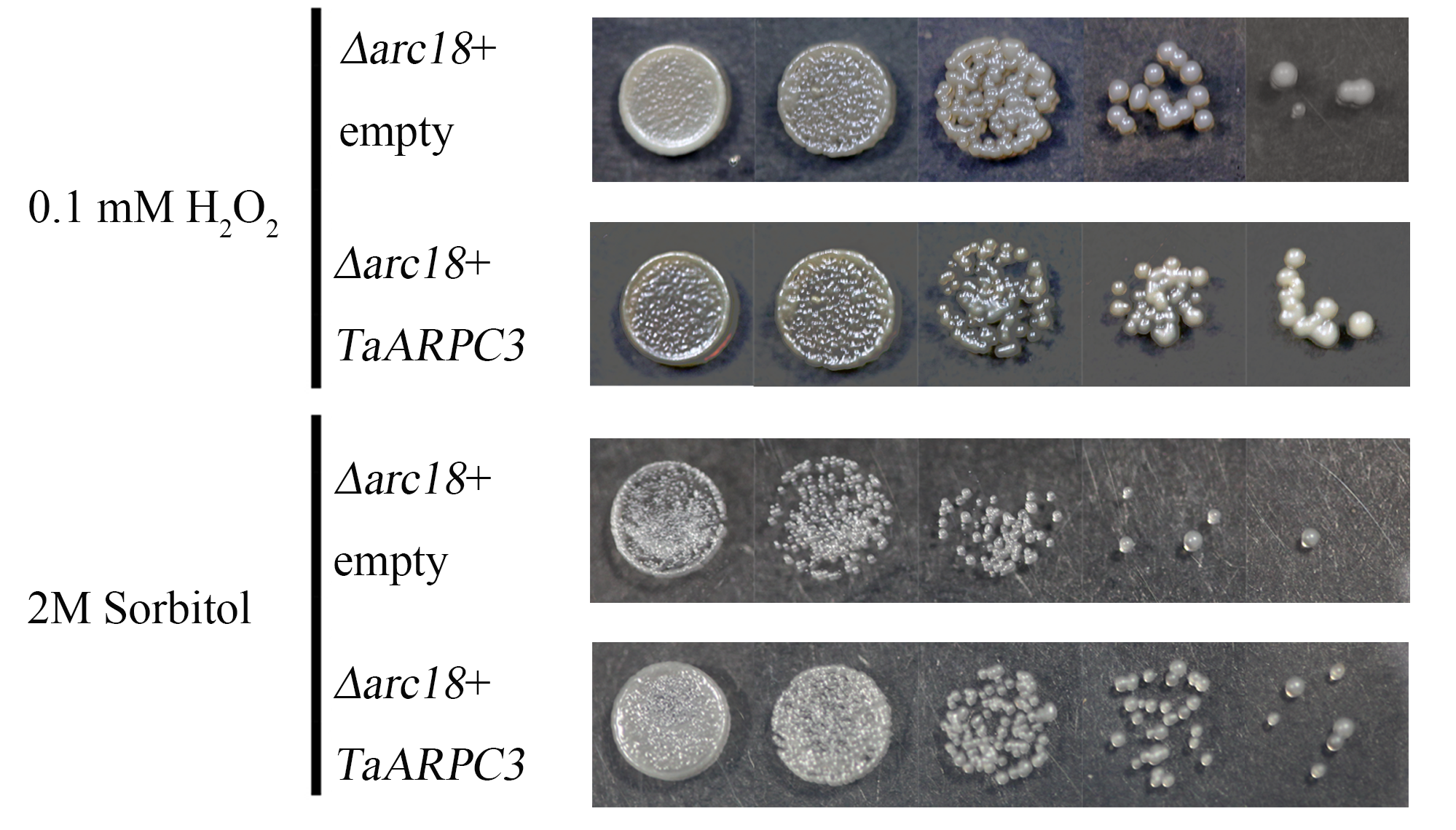

Supplement: FIGURE S2 — Effects of expression of TaARPC3 in yeast cells. 0.1 mM H2O2, solid medium containing 0.1 mM H2O2; 2 M Sorbitol, solid medium containing 2 M Sorbitol. [file Image_2.TIF]
